# Heart Sound Biometric System Based on Marginal Spectrum Analysis

**DOI:** 10.3390/s130202530

**Published:** 2013-02-18

**Authors:** Zhidong Zhao, Qinqin Shen, Fangqin Ren

**Affiliations:** 1 College of Electronics and Information, HangZhou DianZi University, Hangzhou 310018, China; 2 College of Communication Engineering, HangZhou DianZi University, Hangzhou 310018, China; E-Mails: sqq2046@163.com (Q.S.); renfangqin1988@163.com (F.R.)

**Keywords:** heart sound identification system, EEMD, HHT, feature extraction, marginal spectrum

## Abstract

This work presents a heart sound biometric system based on marginal spectrum analysis, which is a new feature extraction technique for identification purposes. This heart sound identification system is comprised of signal acquisition, pre-processing, feature extraction, training, and identification. Experiments on the selection of the optimal values for the system parameters are conducted. The results indicate that the new spectrum coefficients result in a significant increase in the recognition rate of 94.40% compared with that of the traditional Fourier spectrum (84.32%) based on a database of 280 heart sounds from 40 participants.

## 1. Introduction

With the rapid development of transportation, communication, and network technology in modern society, the scope of human activities is broadening, while the difficulty and importance of identification becomes increasingly prominent. Traditional identification methods are generally divided into two kinds: (1) items that people remember, such as user names, passwords, *etc*. and (2) items that people own, such as keys, identification cards, *etc*. The two types both have limitations in that they may be easily forgotten, lost, or imitated. Traditional identification techniques cannot meet the higher security demands of a highly modernized society. Biological recognition technology refers to individual identification based on unique physiological or behavioral characteristics of the human body. Compared with traditional patterns such as password-based or ID card identification, biometrics technology has a high level of safety and reliability, thus making this technology an important international research field of significance in different application areas.

**Figure 1.**
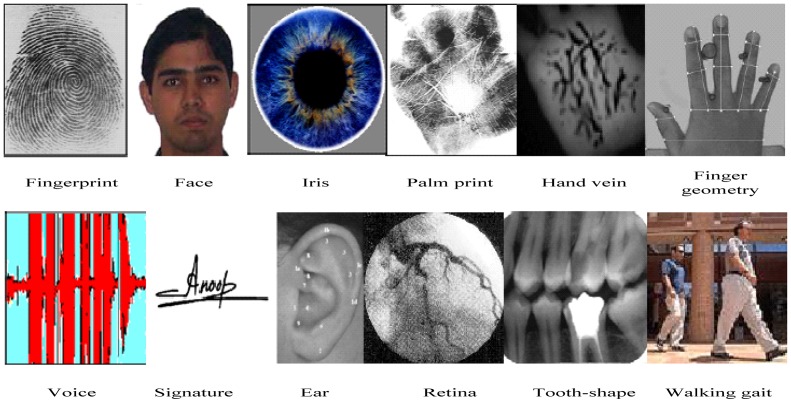
Various biometric features.

As shown in Figure 1, physiological biometrics features commonly include fingerprints, face, hand geometry, eyes (retina and iris), palm prints, ear, tooth, wrist/hand blood-vessel texture, DNA, *etc*. Meanwhile, behavioral biometrics include signature, voice, walking gait, intensity of hitting the keyboard, *etc*. [1,2]. Each biometric technology has its advantages and disadvantages. Jain conducted a simple comparison of different biometrics technologies, and the results are shown in Table 1, where three degrees are used to measure the performance of various biometric features: H stands for high, M for medium, and L for low.

Commercial biometric methods currently include the face, iris, fingerprint, voice, *etc*. These technologies have achieved initial applications, but are faced with many challenges in practical large-scale applications. The most prominent challenge is the issue of biometric security [3]. A fingerprint recognition system was once fooled successfully with fake fingers made of gelatin [4,5]. Moreover, the accuracy of an iris recognition system is also degraded when a printed iris image or a false iris is etched onto contact lenses. Voices could be imitated conveniently, and faces can be easily extracted from the user's photo [6].

Heart sound is the reflection of the mechanical movement of the heart and cardiovascular system. This feature contains physiological and pathological information about the heart and various parts of the body. Compared with previous conventional biometrics features, heart sounds have unique advantages: (1) high security because an individual's heart sounds cannot be faked; (2) easy to process because the heart sound is a one-dimensional signal with frequency components that exist mainly in the low-frequency range, thus making the signal processing simple; (3) high universality because every person has a beating heart. The unique physiological characteristics of heart sounds make them a promising identification technology [7].

**Figure 2.**
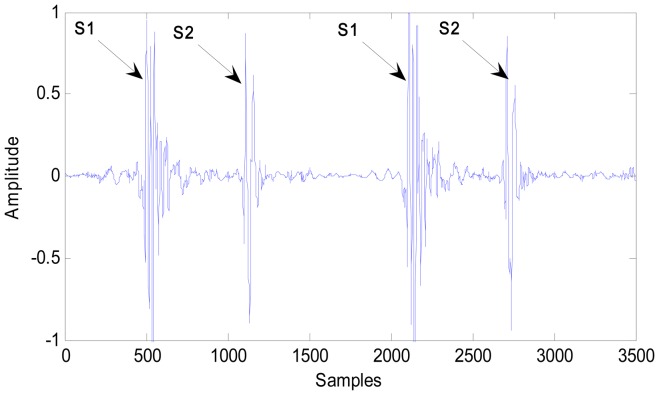
Typical waveform of S1 and S2.

Heart sounds include two parts. The first heart sound (S1) is mainly produced by the closure of the mitral and tricuspid valves. S1 has duration of 70 ms to 150 ms with a frequency of 25 Hz to 45 Hz. The second heart sound (S2) is produced by the closure of the aortic and pulmonary valves. S2 has a duration of 60 ms to 120 ms with a frequency of approximately 50 Hz [7]. A typical waveform of S1 and S2 is shown in Figure 2.

Heart sound identification technology remains at an early research stage, but this technology has been receiving considerable attention. In 2007, Phua from the Singapore Institute of Communication Technology and Beritelli from the Italy University of Catania conducted a preliminary study of heart sound biometrics. One method included cepstrum analysis, followed by the extraction of spectral coefficients, and then the use of the classifiers of the Gaussian Mixture Mode (GMM) and Vector Quantization (VQ) for matching. The system's performance in terms of accuracy identification rate could reach more than 95%, but the experimental sample was inadequate because the total sample size was only 10 [7]. Another method was based on an identification algorithm of heart sound's Fourier spectrum, and the results show that the performance in terms of equal error rate (EER) can be reduced to 9% [8,9].

A large number of studies have recently been developed on this field. In Jasper and Othman's work, wavelet transform (WT) was used to analyze the signal in the time-frequency domain, and then the Shannon energy envelogram was selected as the feature set. Performance was found to be acceptable based on a database of 10 people [10], but as in [7], the sample size was inadequate. Bendary *et al*. [11] extracted three features: auto-correlation, cross-correlation, and cepstrum. The features were used as the feature set. Meanwhile, of the two classifiers used, *i.e.*, mean square error (MSE) and K-nearest neighbor (KNN), KNN was proven to perform better than MSE.

Tao *et al*. used the signals' cycle-power-frequency drawing and improved the D-S information fusion method to realize identity recognition based on heart sounds [12]. Guo *et al*. used a feature set of linear prediction cepstrum coefficient (LPCC), the hidden Markov model (HMM), and wavelet neural network (WNN) to acquire the heart sound classification information and to realize identity recognition [13]. Cheng *et al*. presented a synthetic model of heart sounds and then used the heart sounds' linear band frequency cepstrum (HS-LBFC) as a specified configuration with similarity distance to achieve recognition and verification [14]. The three methods are theoretically feasible but involve feature or model integration that can result in a more complicated implementation of the identification system.

The primary studies on this novel biometric method are summarized in Table 2. Given that no standard database exists and because of the use of different performance metrics, the various performances cannot be compared.

Heart sound biometrics remains at the preliminary research stage with numerous unresolved issues: poor robustness under a noisy environment; the impact of heart diseases on identification accuracy; and non-comprehensive test samples. Meanwhile, accuracy improvement has not yet been explored, given that the auscultation changes in the location of this new biometric technology were mostly borrowed from other biometrics technologies such as speaker identification.

Heart sound are typical non-stationary signals, but traditional signal processing methods such as Fast Fourier Transform (FFT), Short-Time Fourier Transform (STFT), WT, *etc*. cannot easily process heart sounds. Thus, Norden Huang proposed a novel signal processing algorithm called the Hilbert-Huang transform (HHT) [15], which has been widely used in the frequency analysis of non-stationary signals and has been proven to be a powerful tool for non-stationary signal processing.

In this paper, a method for the extraction of a novel feature, the marginal spectrum used in identification based on heart sounds, is presented. HHT consists of two main parts: Empirical Mode Decomposition (EMD), which can be replaced by Ensemble Empirical Mode Decomposition (EEMD), and Hilbert transform (HT). The basic procedure when using HHT to extract a non-stationary signal's spectrum is as follows: first, the given signal is decomposed into several intrinsic mode functions (IMFs) by EEMD. HT is then applied to each IMF to obtain the corresponding Hilbert spectrum. That is, each IMF is expressed in the time-frequency domain, and then all the IMF's Hilbert spectrum will be aggregated to derive the original signal's Hilbert spectrum. Finally, the signal's marginal spectrum is derived in the feature extraction stage.

The remainder of this paper is organized as follows: Section 2 presents the theory of EMD, EEMD, and marginal spectrum. Section 3 describes the heart sound identification system, especially the extraction steps, in detail. The optimal system parameters are illustrated in Section 4 by experiments. Finally, Section 5 summarizes our conclusions.

## 2. Basic Theory of HHT and Its Marginal Spectrum

### 2.1. EMD

Most non-stationary signals may contain more than one oscillation mode, which is why the HT of the original signal cannot produce an accurate instantaneous frequency of non-stationary signal. Empirical mode decomposition can adaptively decompose signal into a finite and often a series of small number of IMFs, which satisfy the following two conditions:

1. In the whole signal, the number of extrema and zero-crossings must be equal to or less than one.
2. At any point in the signal, the mean value of the envelope defined by the local maxima and that defined by the local minima must be zero.

These two conditions guarantee the well-behaved HT. The IMFs represent the oscillatory modes embedded in signal. Applying HT to each IMF, the instantaneous frequency and amplitude of each IMF can be obtained which constitute the time-frequency-energy distribution of signal, called as Hilbert spectrum. Hilbert spectrum provides higher resolution and concentration in time-frequency plane and avoids the false high frequency and energy dispersion existed in Fourier spectrum.

To obtain meaningful instantaneous frequency, the signal must be decomposed into a number of different IMFs by sifting process, which can be separated into the following steps:

1. All the local extrema are identified, and then all of the local maxima are connected by a cubic spline line as the upper envelope.
2. Similar to step1, all the minima are connected by a cubic spline line as the lower envelope.
3. The mean of the upper and lower envelopes is defined as *m*_1_, which is removed from the original signal *x*(*t*) to obtain *h*_1_:
  (1) $$h_{1}=x(t)-m_{1}$$
  *h*_1_ should be an IMF because the construction of *h*_1_ satisfies all the above IMFs' conditions. However, in the case of a dramatic change in the original signal, the cubic spline line often generates new extrema or moves the pre-existing extrema. Moreover, the cubic spline line still has some problems at both ends of the signal, and the boundary effect reduces the accuracy of the IMFs' construction process. Thus, we need to repeat the above steps to obtain an exact IMF, that is,
4. When *h*_1_ does not meet IMF's conditions, step (1) to (3) are repeated to obtain the mean envelope *m*_11_, and then *h*_11_ = *h*_1_ − *m*_11_ are used to determine whether the IMFs' conditions are met. This process can be repeated up to k times to obtain *h*_1_*_k_* as an IMF. Designated as *c*_1_ = *h*_1_*_k_*, *c*_1_ denotes the first IMF component of the signal.
5. *c*_1_ is subtracted from the original signal, and the difference is the residue component *r*_1_:
  (2) $$r_{1}=x(t)-c_{1}$$
  where *r*_1_ is taken as the new original signal, and the second IMF component *c*_2_ can be obtained by using the above process. Thus, the above process is repeated n times to obtain n IMF components of the signal:
  (3) $$\left\{ \begin{array}{c} r_{1}-c_{2}=r_{2}\\ r_{2}-c_{3}=r_{3}\\ .\\ .\\ .\\ r_{n-1}-c_{n}=r_{n} \end{array}\right.$$

Eventually, the stopping rule is: (1) the component *c_n_* or the residue component *r_n_* is less than a predetermined threshold or (2) the residue component *r_n_* becomes a monotonic function, such that a new IMF cannot be obtained from it. Finally, the signal can be expressed as the sum of n IMFs and the residual component:

(4) $$x(t)=\sum_{i=1}^{n}c_{i}+r_{n}$$

where *c*_1_, *c*_2_,…, *c_n_* represent the signal characteristics in the different time scales. These IMFs contain the signal of different frequency components from high to low, and each IMF includes a frequency band range that adaptively changes with a specific signal, which is one of the advantages of EMD. EMD does not use any pre-determined filter or function, which is a data driven method.

### 2.2. EEMD

Although EMD has numerous advantages in terms of the analysis of non-stationary signals, most signals are not always symmetrically distributed on the timeline, which may result in a condition wherein one IMF includes large-scale differences in signal components or wherein similar components exist in different IMFs. This condition is called as the mode aliasing phenomenon, which greatly affects signal analysis. To solve the mode mixing problem, a new method called EEMD was proposed by Huang in 2009 [16]. By using the Gaussian white noise's characteristics of regular distribution in the frequency domain, this method adds Gaussian white noise into the analyzed signal to make such signal continuous at different scales to avoid mode mixing.

The principle of EEMD is very simple. All the processed signals can be decomposed into two parts: signal and noise. Each signal is independently observed to contain different noises. To provide a regular and relevant distribution size, white Gaussian noise is added to the signal. The additional noise will reduce the signal-to-noise ratio (SNR) and can help eliminate the aliasing mode. When the Gaussian white noise of regular distribution is added to the signal, different scales of the signal are automatically decomposed into the appropriate scales associated with the Gaussian white noise. Finally, the decomposition results are averaged to eliminate the added noise. The steps are shown as follows:

A mean of zero standard deviation of a constant Gaussian white noise *w*(*t*) is added to the processed signal *x*(*t*) to obtain an new signal *X*(*t*), that is, *X*(*t*) = *x*(*t*) + *w*(*t*). EMD is applied to *X*(*t*) to obtain each IMF component *c_j_* and the residual component *r_n_*:

(5) $$X(t)=\sum_{j=1}^{n}c_{j}+r_{n}$$

Different white noises *w_i_*(*t*) are added to the processed signal to obtain *X_i_*(*t*) = *x*(*t*) + *w_i_*(*t*). The above steps are repeated to obtain the IMF's component:

(6) $$X_{i}(t)=\sum_{i=1}^{n}c_{ij}+r_{in}$$

According to the principle that an unrelated random sequence's statistic mean is zero, the corresponding IMF's mean value is believed to eliminate the impact of the added Gaussian white noise, and the final IMFs is:

(7) $$c_{j}=\frac{1}{N}\sum_{i=1}^{N}c_{ij}$$

where N denotes the total ensemble number.

EEMD takes advantage of the statistical properties of noise and the decomposition principle of EMD, thus making it a suitable binary filter for arbitrary data. Model aliasing can also be solved effectively by adding white noise.

In addition, EEMD decreases the amount of the noise based on the statistical law of the following formula [16]:

(8) $$\hat{\varepsilon }=\frac{\varepsilon }{\sqrt{N}}$$

where *N* is the overall number, *ε* is the magnitude of the noise; and *ε̂* is the error between the processed signal and the final IMFs, which are summed to obtain signal. In other words, in the case of constant noise amplitude, a greater overall number *N* yields a more accurate final result of decomposition. However, if the amplitude is extremely small and the SNR is extremely high, the noise would not affect the extremas' selection, thus losing the role of supplementary scales.

**Figure 3.**
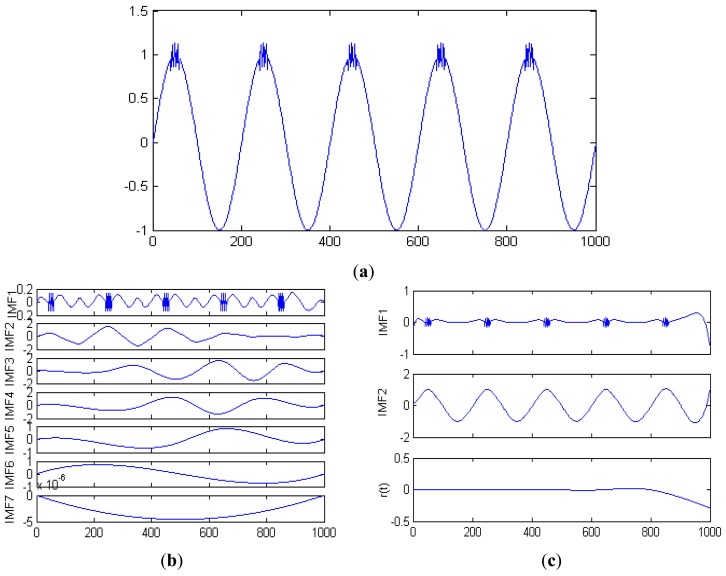
(**a**) Simulation signal; (**b**) IMFs of the simulation signal obtained by using EMD; (**c**) IMFs of the simulation signal obtained by using EEMD.

Supposing the simulation signal's expression is:

(9) $$\begin{array}{l} \begin{array}{cc} a=\sin(2\times \pi \times t)+10\times w(t)*\delta (t-n) & \left(n=...,-2,-1,0,1,2,...\right) \end{array}\\ w(t)=\{\begin{array}{cc} t-0.2-0.015\times m, & 0.2+0.03\times m<t<0.215+0.03\times m\\ 0.215+0.015\times m-t, & 0.215+0.03\times m<t<0.23+0.03\times m \end{array}\\ m=0,1,2,3 \end{array}$$

where the signal point is 1,000, and sampling frequency fs is 2,000 Hz. Figure 3(a) shows this signal. After being decomposed by EMD, the IMFs are shown in Figure 3(b). We select N as 100 and the simulation signal is processed by using EEMD. The previous two IMFs and the residue component r(t) are shown in the Figure 3(c).

The similar components are evident in different IMFs in Figure 3(b) but are not present in Figure 3(c). The mode aliasing phenomenon is effectively eliminated by using EEMD.

**Figure 4.**
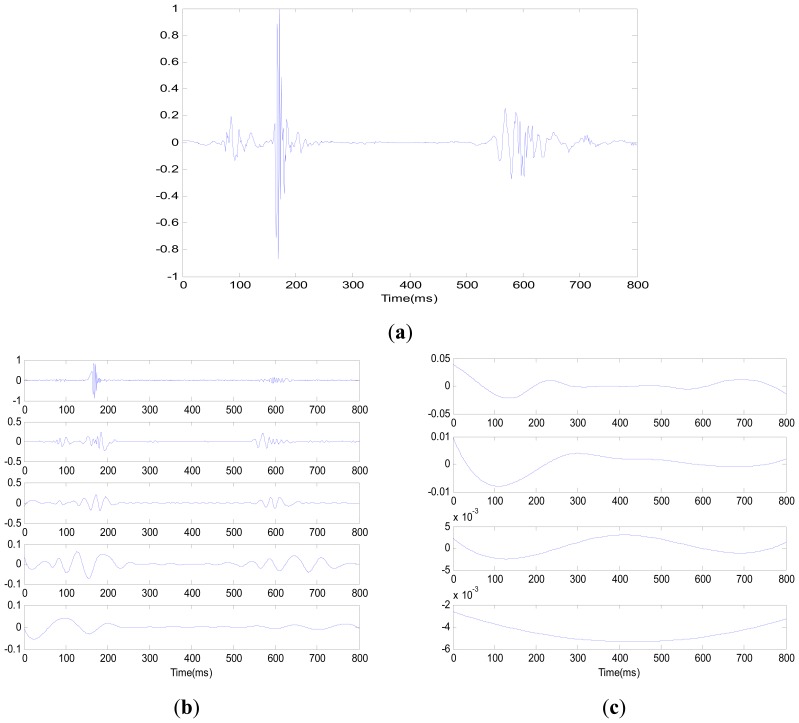
(**a**) Cardiac cycle of a heart sound signal; (**b**) First five IMF components of (**a**)'s signal; (**c**) Last four IMF components of (**a**)'s signal.

Figure 4(a) is a cardiac cycle of a heart sound signal. Each IMF is shown in Figure 4(b,c) by EEMD. The signal is sequently decomposed into the highest frequency component to the lowest frequency component. The decomposition basic function that is directly obtained from the signal is adaptive, which guarantees the inherent characteristic of signal and avoids the diffusion and leakage of signal energy. This feature is a significant improvement of EEMD compared with traditional signal processes. For the Fourier transform, the decomposition basic function is a fixed frequency and amplitude harmonic wave function, where each harmonic wave function characterizes the energy at a specific frequency.

For the wavelet and wavelet packet analyses basis functions are determined in advance and do not change with different signals. Obviously, different decomposition basic functions are needed for the optimal decomposition of different signals. The best results cannot be guaranteed with pre-determined basic functions.

EEMD's adaptability can characterize the properties of the signal, thus ensuring the accuracy of decomposition. Moreover, each IMF component shows a clear physical meaning, which enables the derivation of instantaneous frequency after HT. Signal frequency can be expressed precisely, which is why EEMD is suitable for non-stationary signal processing.

### 2.3. Hilbert Spectrum and Hilbert Marginal Spectrum

For a given signal *x*(*t*), the HT is defined as:

(10) $$y(t)=H[x(t)]=\frac{1}{\pi }\int_{-\infty }^{\infty }\frac{x(\tau )}{t-\tau }d\tau$$

and the analytic signal *z*(*t*)is:

(11) $$z(t)=x(t)+jy(t)=a(t)e^{j\varphi (t)}$$

*a*(*t*) = [*x*^2^(*t*) + *y*^2^(*t*)]^1/2^ is the instantaneous amplitude, and *φ*(*t*) = arctan(*y*(*t*)/*x*(*t*)) is the instantaneous phase. The instantaneous frequency *ω*(*t*) can be given by:

(12) $$\omega (t)=\frac{d\varphi (t)}{dt}$$

Having obtained the IMFs by using EEMD method, HT can be applied to each IMF component, and each IMF component can be expressed as:

(13) ci(t)=Re(ai(t)ejφi(t))

With the instantaneous frequency, each IMF can also be expressed as:

(14) ci(t)=Re(ai(t)ej∫ωi(t)dt)

Thus, the original signal can be expressed in the following form:

(15) x(t)=Re(∑i=1nai(t)exp(j∫ωi(t)dt))

where the residue *r_n_* is discarded.

Meanwhile, for the same signal *x*(*t*), the Fourier expansion can be expressed as:

(16) $$x(t)=\sum_{i=1}^{\infty }a_{i}e^{j\omega_{i}t}$$

where *a_i_* and *ω_i_* are both constants.

From Equations (15) and (16), it shows that the Fourier transform is a special form of the HT. Amplitude variation and instantaneous frequency not only improve the effectiveness of decomposition significantly, but also make HHT suitable for non-stationary signals. The transformations of amplitude and frequency can be clearly separated by using each IMF component's expansion, which mitigates Fourier transform's limitation in terms of invariable amplitude and frequency. The time-frequency-amplitude distribution is designated as the signal's Hilbert spectrum *H*(*ω,t*), which can accurately describe amplitude changes with time and frequency and can further reflect the signal's inherent time-varying characteristics.

The marginal spectrum *H*(*ω*) can be defined as:

(17) $$H(\omega )=\int_{0}^{T}H(\omega ,t)dt$$

**Figure 5.**
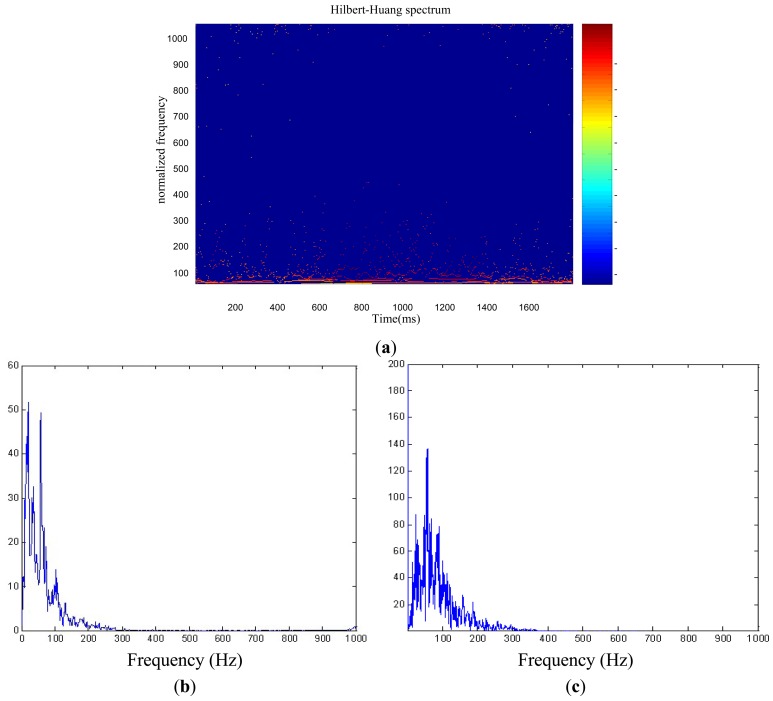
(**a**) Hilbert spectrum of the cardiac cycle; (**b**) Marginal spectrum of the cardiac cycle; and (**c**) Fourier spectrum of the cardiac cycle.

The Hilbert spectrum offers a measure of amplitude contribution from each frequency and time, whereas the marginal spectrum offers a measure of the total amplitude (or energy) from each frequency [15]. The Hilbert spectrum and the marginal spectrum are shown separately in Figure 5(a,b). Figure 5(c) shows the Fourier spectrum.

Figure 5(a) provides distinct information on the time-frequency contents of the cardiac cycle of an object, which clearly reveals the dynamic characteristic of the cardiac cycle in the time-frequency plane. The marginal spectrum shown in Figure 5(b) represents the fluctuation of the energy distribution of heart sound with frequency, which mainly has two frequency pikes. The first pike lies at approximately 20 Hz to 40 Hz, which corresponds to the frequency range of S1. The second pike lies at approximately 80 Hz to 120 Hz, which corresponds to the frequency range of S2.

Figure 5(c) is the Fourier spectrum of the cardiac cycle. Comparing to the marginal spectrum, the Fourier spectrum shows rich higher frequency components, *i.e.*, the harmonic components. These appeared additional harmonic components are used to simulate nonstationary heart sound. As a result, the harmonic components divert energies to a much wider frequency domain. Both marginal spectrum and Fourier spectrum reflect signal energy changing with frequency, but the Fourier spectrum does not provide reasonable physical explanations since the data is not stationary.

Based on the accuracy of signal decomposition and on the advantages of HHT, the marginal spectrum is used in this paper and is compared with the Fourier spectrum in the experiments in Section 4, which will prove that the marginal spectrum can be an effective representation of a heart sound's personality trait.

## 3. Heart Sound Identification System

**Figure 6.**
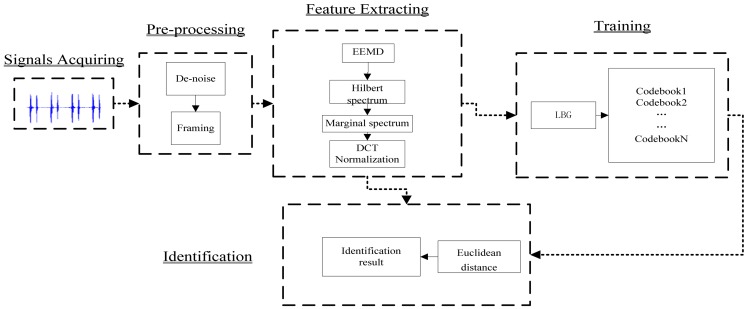
Block diagram of heart sound identification system.

This novel identification system comprises five parts: signal acquisition, pre-processing, feature extraction, training, and identification. Figure 6 shows a block diagram of this identification system.

### 3.1. Signals Acquisition

**Figure 7.**
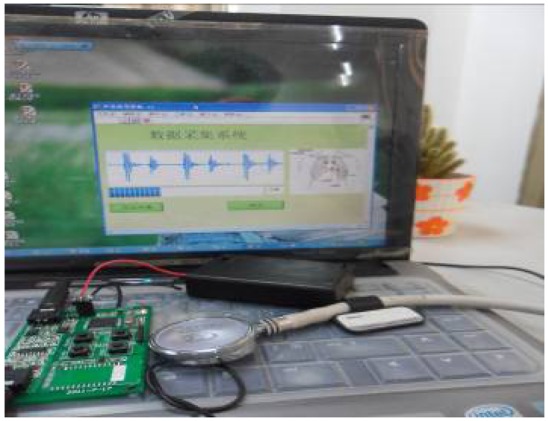
Equipment for signal acquisition.

The use of a computer's sound card can facilitate the acquisition of signals with high accuracy and medium sampling frequency. Before signal acquisition, the sampling frequency, sampling bits, and buffer size should be set. In this work, the equipment for signal acquisition shown in Figure 7 and the personal computer's sound card is used to pick up heart sound signals. Given that the heart sound frequency compositions mainly concentrate in the low frequency domain and that the highest frequency component does not exceed 200 Hz, we choose a sampling frequency of 2,000, a sampling bit of 16, and a buffer size of 4,000. During signal acquisition, participants are required to be calm and relaxed. A digital stethoscope is placed on the participants' chest in the pulmonary auscultation region. The recorded heart sound database comprises 280 heart sounds from 40 participants, which last approximately 10 s. The interval between each recorded signal is at least an hour. The location on the chest for all recordings is same.

### 3.2. Pre-Processing

#### 3.2.1. De-Noising

Given the influence of the acquisition environment and of the electronic stethoscope, the collected heart sound signals contain various kinds of noises, which is still affected by interferences such as body movement, lung sounds and other surrounding sounds. If these noises are not removed, the accuracy of the features will be seriously affected and ultimately influence the identification system's performance. Given that heart sounds are non-stationary, the discrete WT (DWT) was utilized to solve this problem.

**Figure 8.**
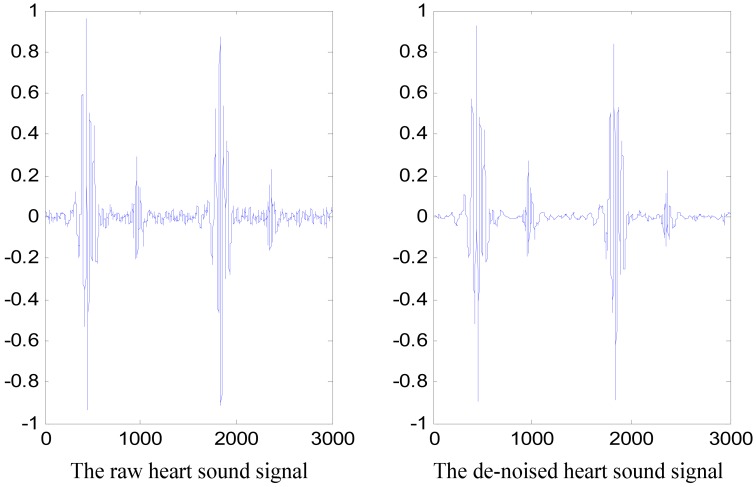
Raw heart sound signal and the corresponding de-noised signal.

The specific de-noising steps are: (1) the fifth-order Daubechies wavelet is selected as the mother wavelet to decompose the heart sound signal into six scales; (2) the WT coefficients at the third, fourth, fifth, and sixth scales are retained based on an energy-based threshold [17], whereas the coefficients related to other scales are set to zero; and (3) the signal is reconstructed by using inverse DWT (IDWT). A de-noised heart sound signal along with its corresponding raw signal is depicted in Figure 8.

#### 3.2.2. Framing

Given the non-stationary nature of heart sounds, signals must be divided into short segments called frames. Obviously, the subsequent feature extraction is based on each frame. The length of this segment is called frame length, and the distance from the beginning of a frame to the beginning of the subsequent one is called frame shift. Numerous windows can be applied to decompose signals into frames. The duration of the window is equal to the frame length. In the experiment phase, we will discuss whether different types of windows as well as different frame lengths and frame shifts would affect the system's performance.

### 3.3. Feature Extracting

**Figure 9.**
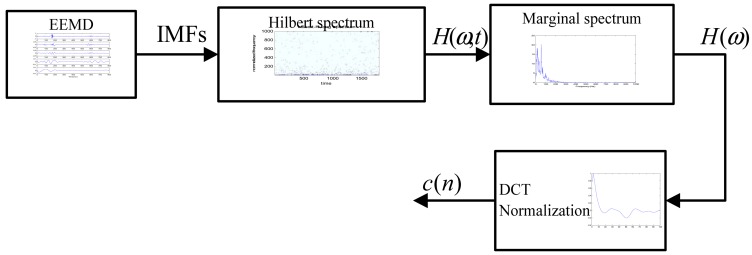
Block diagram of feature extraction process.

In this work, the marginal spectrum is extracted as the feature. Figure 9 shows the flow chart of the feature extraction procedure.

1. EEMD is applied to each frame signal *x*(*n*) to obtain the IMFs of the signal. Then, two parameters: the number of the ensemble *N* and the ratio between the added noise's standard deviation and the heart sound *r* are derived. Two experiments will be conducted to select an optimal set for *N* and *r*.
2. Hilbert spectrum *H*(*ω*,*t*) of each frame will be determined by applying HT to each IMF, which represents the amplitude and the instantaneous frequency in a three-dimensional plot with respect to time and reveals clearly the physiological properties of heart sound in time-frequency plane.
3. Three-domain Hilbert spectrum *H*(*ω*,*t*) of each frame signal is integral in the time domain to obtain the Marginal spectrum *H*(*ω*). The performance will be compared with that of the Fourier spectrum, which has the same physical meaning.
4. Dimension compression and amplitude normalization: the spectrum coefficients *c*(*n*) are followed by the Discrete Cosine Transform (DCT). The dimension is reduced to an appropriate dimension for the subsequent training and identification phase. In the experiment phase, the effects of different dimensions and of amplitude normalization will be used to normalize the spectrum coefficient amplitudes to a range between 0 and 1 by dividing the absolute maximum value of these coefficients.

### 3.4. Training

VQ is a conventional and successful classifier in pattern recognition [18]. Compared with other identification models (such as GMM), VQ model's advantage lies in the simplicity of the design and little calculation time, which is very suitable for quick recognition. So in this work, VQ is used as a classifier in the heart sound identification system. The basic idea behind VQ is to compress a large number of feature vectors into a small set of code vectors. Given a vector source with known statistical properties, a distortion measure, and a given number of code-vectors, a codebook and partition need to be identified to achieve the smallest average distortion. Assuming the smallest average distortion is given by:

(18) $$D_{i}=\frac{1}{T}\sum_{j=1}^{T}\underset{1\leq m\leq M}{\min}\left[d(x_{j},{\text{B}}_{m}^{i})\right]$$

where *x_j_*(*j* = 1,2,…,*T*) is the feature vector arising from the user who will be identified. ${\text{B}}_{m}^{i}$ is the codebook formatted in the training phase, and it presents the i-th codebook of m-th codeword. For $d(x_{j},{\text{B}}_{m}^{i})$, the Euclidean distance can be used for measurement. In the identification stage, each user's *D* is calculated by using Equation (18), and the final recognition result is the smallest *D* that corresponds to the person.

In these experiments, the VQ codebook is trained with the Linde-Buzo-Gray algorithm [19] iteratively to minimize the quantization error, and the Euclidean distance is used to measure the quantization error. LBG algorithm bypasses the need for multi-dimensional integration. In this work, we use LBG-VQ algorithm for the proposed heart sound based identification. We will determine the optimal choice of the number of mixture components for the VQ later.

## 4. Experiments Results and Analysis

In this system, the recognition method can be classified as one-to-many comparisons. That is, all the users' templates in the database must be searched for a match when an individual is recognized. The decision criterion is the minimum Euclidean distance described above. Three signal recordings of each participant are used in this phase to test the system's performance. The results of these experiments will be described based on the correct recognition rate (CRR):

(19) $$\text{CRR}=\frac{C_{n}}{T_{n}}\times 100$$

where *C_n_* is the number of correct recognitions, and *T_n_* is the total number of testing samples. That is, CRR is our system's performance metric.

There are 40 participants and 7 heart sound recordings for each participant. Each recording lasts approximately 10 s. Four recordings of each person are selected randomly to build the model in the training phase. The rests are randomly used for identification in the testing phase. Heart sound biometric system based on marginal spectrum analysis and VQ presented in Section 3 is used. Preliminary experiments are conducted to evaluate the performance of different parameter setting: EEMD parameters, number of VQ codebook, Window type, frame length and frame shift, DCT dimension compression degree and different feature sets.

### 4.1. Window Type, Frame Length and Frame Shift

In this section, the effects of different window types, frame lengths, and frames shifts will be discussed. According to [7], a standard speech frame length of 20 ms to 25 ms is inappropriate for heart sounds because of their quasi-periodic nature. Thus, the frame length should be longer than 20 ms to 25 ms. In [7], when frame shift is equal to the frame length, that is, no overlap exists between frames, the performance is optimal. In this system, the signal lengths are appropriate, such that tests on non-overlap property are not needed to determine whether the tool remains suitable.

First, the window type is changed on the basis of a fixed the frame length of 256 ms and frame shift of 64 ms, VQ-32, feature dimension of 100, N of 20 and r of 0.2. As shown in the Table 3, when applying the Hamming window, the CRR is the highest. However, this influence is minimal because the Hamming window is superior to the Hanning window by only 1.66% in terms of CRR. The following conclusion can be drawn: window type does not significantly affect system performance.

**Figure 10.**
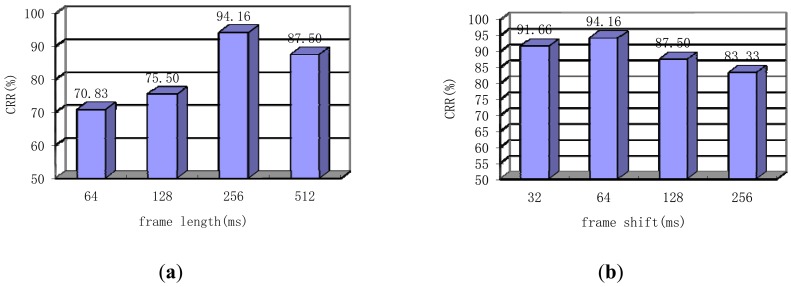
(**a**) Performance of the system when using different frame lengths; (**b**) Performance of the system when using different frame shifts.

Next, the CRR of our system is evaluated based on different frame lengths with fixed Hamming window type and a frame shift of a quarter frame length. Note that, the other parameters also choose the optimal parameters: VQ-32, feature dimension of 100, N of 20 and r of 0.2. The frame length was increased from 64 ms to 512 ms. the conclusion of the longer segment is confirmed in Figure 10(a). A frame length of 256 ms is the optimal choice. If conditions remain constant, the heart sound is usually considered a quasi-stationary signal for a longer time than speech signal, the frame length of which is usually about 20–25 ms. Finally, the influence of frame shift based on a Hamming window type and a frame length of 256 ms is verified. Frame shift was increased from 32 ms to 256 ms. The results shown in Figure 10(b) indicate that when the frame shift is a quarter frame length, the best performance is achieved. This conclusion differs from that of [7], where the best results are achieved with the non-overlap property. Moreover, this finding proves that non-overlapping of a few frames is not optimal in cases with insufficient signal length. The result does not conflict with that of [7], which conforms to different parameters.

### 4.2. Performance under Different EEMD Parameters

To use EEMD for the calculation of the marginal spectrum, two parameters must be predetermined: the number of the ensemble N and the ratio between the added noise's standard deviation and that of the heart sound r.

**Figure 11.**
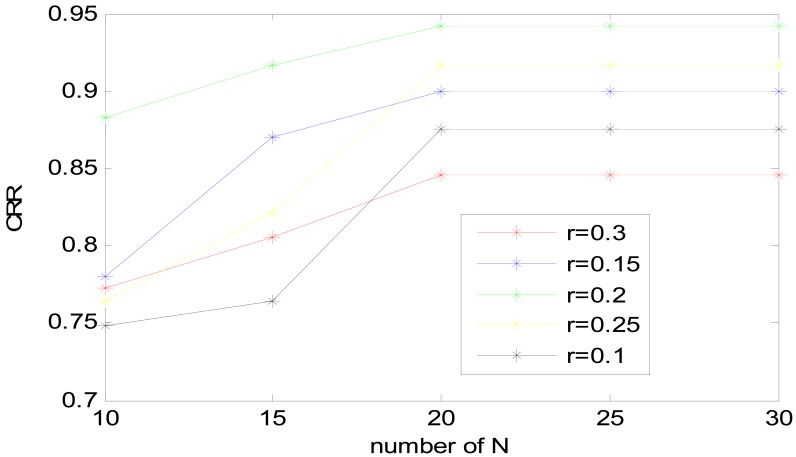
Performance of the heart sound identification system based on EEMD's *N* and *r*.

Experiment was performed to select an optical set for the accurate extraction of spectrum features as the new feature set. Note that, a frame length of 256 ms with frame shift of 64 ms, VQ-32, Hamming window type and feature dimension of 100 have been used. We will discuss the choice of these parameters later. In order to determine to the optimal N and r value, N was chosen from 10 to 30 by 5 steps, r varied from 0.1 to 0.3. The results of CRR based on two parameters are shown in Figure 11.

As shown in Figure 11, when r is 0.2 and N is 20, the heart sound identification system achieves the best result of 94.16%. However, when the value of r is fixed, a greater N does not necessarily result in better performance. When N reaches a certain value 20, the precision of decomposition is at its maximum, and increasing N would no longer improve decomposition accuracy. Because EEMD improves the uniform distribution of signals, too much white noise is useless when signals have reached the uniform distribution.

Similar to the previous analysis about r, if the noise is extremely small, it may not cause the change of extrema, thus losing the role of supplementary scales and ultimately affecting the results of feature extraction. If the added noise is extremely large, the added noise will affect the SNR and confuse the decomposition results. So r was fixed 0.2 as a middle value with N = 20, which was the optimal choice for this system.

### 4.3. DCT Dimension Compression Degree

**Figure 12.**
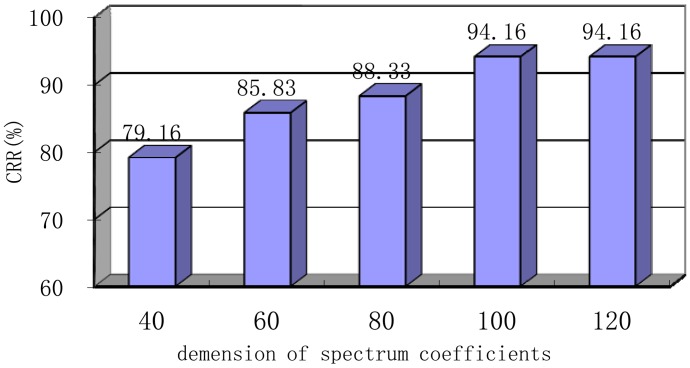
Performance based on the different DCT dimension compression degree with frame length of 256 ms, frame shift of 64 ms, N of 20, r of 0.2, VQ-32 and Hamming window.

Spectrum coefficients are transformed by using DCT, and the energy can be concentrated on a few transform coefficients. Dimension compression could be easily realized through the removal of the smaller energy contribution coefficient while retaining the bigger ones. DCT not only avoids the obvious distortion of the original spectrum coefficients but also achieves dimension compression. In this section, spectrum coefficients are compressed from 40 to 120 to test the influence of DCT compression degree on the CRR at an original dimension of 256 based on VQ-32, Hamming window type, frame length of 256 ms, frame shift of 64 ms, N of 20 and r of 0.2. As shown in Figure 12, this system has achieved the best CRR of 94.16% with 100 spectrum coefficient dimension. When the dimension is compressed to be extremely small, the processed coefficients can no longer represent the raw spectrum. Accordingly, system performance will be degraded. At 40 spectrum coefficient, the CRR is only 79.16%. With decreasing compression degree, the CRR increases because of the lower distortion rate. When the dimension increased up to 100, the CRR can no longer increase.

### 4.4. Number of VQ Codebooks

**Figure 13.**
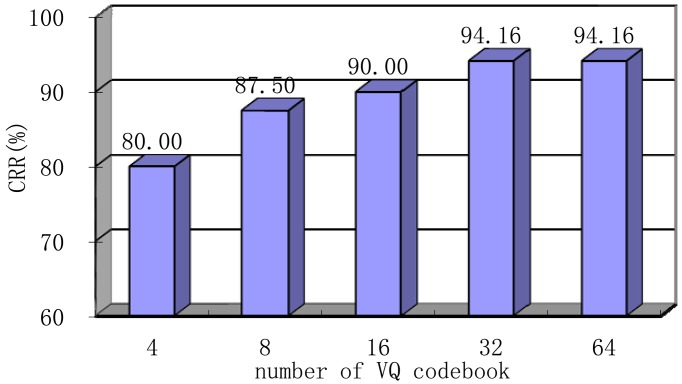
Performance of the system based on the number of codebooks with frame length of 256 ms, frame shift of 64 ms, N of 20, r of 0.2, feature dimension of 100 and Hamming window.

In this section, we determine the optimal number of codebooks for VQ when N and r are kept constant 20 and 0.2, respectively. Note that, Hamming window type, feature dimension of 100, frame length of 256 ms, and frame shift of 64 ms have also been chosen. We will discuss the choice of these parameters later. As shown in Figure 13, this VQ-based system reaches its best performance at a codebook of 32 (VQ-32). When the codebook number is extremely small, the original spectrum coefficients cannot be accurately approximated. Meanwhile, if the number is great, more codebooks require more storage space, and the computational complexity will also increase significantly. Considering the factors above, VQ-32 is selected as the final training model in the following section.

**Figure 14.**
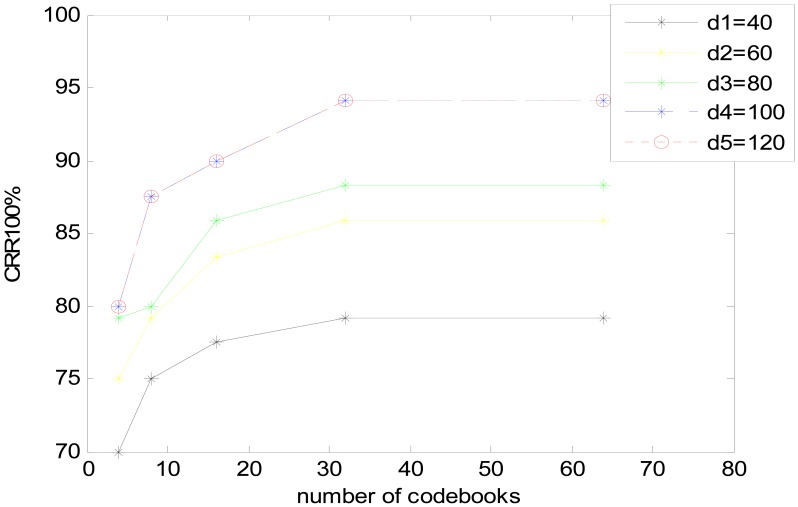
Performance of the heart sound identification system based on different DCT dimension and codebooks.

Furthermore, another experiment is made to validate the optimal DCT dimension compression degree and Number of VQ codebook, DCT dimension compression degree is chosen as 40, 60, 80, 100, 120 and number of VQ codebook is chosen as 4, 8, 16, 32, 64. The results of CRR based on two parameters are shown in Figure 14.

DCT dimension compression degree-100 and VQ-32 is the optimal choice for this system. The result accord with the above experiments separately.

### 4.5. Marginal Spectrum and Fourier Spectrum

In this work, the marginal and Fourier spectra, which have the same physical feature sets, are used. During the extraction of the marginal spectrum, N of 20, r of 0.2, VQ-32, Hamming window type, feature dimension of 100, frame length of 256 ms, and frame shift of 64 ms have been used as the optimal values discussed above. Results are shown in Table 4.

Table 4 shows that the improvement in the CRR when the marginal spectrum is used as the feature set. A CRR increase of up to approximately 10% can be achieved. The improvement can be attributed to the marginal spectrum's better characterization of non-stationary heart sound compared with the Fourier spectrum, as demonstrated in Section 2.

### 4.6. Performance using Different Database

Identification performance with respect to another database is discussed in this section. An open heart sounds database HSCT-11 collected by the University of Catania Italy is adopted to evaluate the performance of heart sounds biometric systems, which is a collection of heart sounds to be used for research purpose in the field of heart-sounds biometry and freely available at the address. It contains heart sounds acquired from 206 people, *i.e.*, 157 male and 49 female [20,21]. Eighty people (65 male and 15 female) are selected randomly in our experiments. N of 20, r of 0.2, VQ-32, Hamming window type, feature dimension of 100, frame length of 256 ms, and frame shift of 64 ms have been used as the optimal values discussed above. The performance of the biometrics system is expressed in terms of CRR, which achieves 92%. It is clear that the results are also very encouraging across the larger database.

## 5. Conclusions

This paper proposed a new biometric method based on heart sounds by using a novel feature set, the marginal spectrum *versus* a normal feature of the Fourier spectrum. In the experimental phase, the optimal value of two parameters in EEMD method is first determined, and then the number of VQ codebooks is set. The DCT dimension compression degree was also considered. Finally, the effects of window type, frame length, and frames shift were tested. After parameters were set to the optimal values: *N* of 20, *r* of 0.2 in the EEMD method, VQ-32, Hamming window type, feature dimension of 100, frame length of 256 ms, and frame shift of 64 ms, the feature set of marginal spectrum was compared with that of the Fourier spectrum. The marginal spectrum, which is suitable for non-stationary signal processing, achieved a CRR of 94.16% in our identification system, whereas the CRR was 84.93% for the Fourier spectrum in the same experimental environment. The marginal spectrum was found to be a better feature set than the Fourier spectrum in the heart sound biometric system.

This approach may achieve improvements in identification performance compared with systems proposed in the related literature, and the marginal spectrum can undeniably be used as a novel feature set in heart sound biometric systems, and the experiment sample is far larger than that in other studies [7–10].

Future work would be directed towards the use of different classification schemes such as the GMM to build a different identification system that can prove this novel feature's applicability and superiority. Meanwhile, other factors that affect the result such as different de-noising methods, *etc*., must be considered.

The results indicated that heart sounds could be considered a promising biometric technology. Although the proposed system was found to achieve good performance under the experimental conditions, numerous factors have to be considered in practical applications, such as signal stability and a large signal database ccontaining a large number of peoples with varying age, conditions, emotion and diversified heart disease. Thus, future work will also have to concentrate on the two aforementioned aspects to improve this novel biometric method.

## Figures and Tables

**Table 1.** Comparison of various biometric technologies' performance.

**Biometrics**	**Universality**	**Distinctiveness**	**Permanence**	**Collectability**	**Feasibility**	**Acceptability**	**Circumvention**
Hand vein	M	M	M	M	M	M	L
Walking gait	M	L	L	H	L	H	M
Odor	H	H	H	L	L	M	L
Ear	M	M	H	M	M	H	M
Finger geometry	M	M	M	H	M	M	M
Fingerprint	M	H	H	M	H	M	M
Face	H	L	M	H	L	H	H
Retina	H	H	M	L	H	L	L
Iris	H	H	H	M	H	L	L
Palm print	M	H	H	M	H	M	M
Voice	M	L	L	M	L	H	H
Signature	L	L	L	H	L	H	H
DNA	H	H	H	L	H	L	L

**Table 2.** Primary studies on heart sound biometrics.

**Author**	**Year**	**Database Size**	**Feature Set**	**Classification**	**Identification Mode**
Phua *et al*.	2007	10 people	LBFC MFCC	VQ GMM	Recognition
Beritelli *et al*.	2007	20 people	STFT	Euclidean distance	Verification
Jasper *et al*.	2010	10 people	Envelogram	Euclidean distance	Recognition
Bendary *et al*.	2010	40 people	Auto-correlation, Cross-correlation Cepstrum	MSE KNN	Recognition
Tao *et al*.	2010	5 to 100 people	Cycle Power Frequency	-	Verification Recognition
Guo *et al*.	2010	80 people	LPCC	HMM WNN	Recognition
Cheng *et al*.	2012	12 people	HS-LBFC	similarity distance	Verification Recognition

**Table 3.** CRR when using different window types.

**Window Type**	**CRR (%)**
Rectangular window	93.33
Hamming window	94.16
Hanning window	92.50

**Table 4.** The CRR using different feature set.

**Different feature set**	**CRR(%)**
Marginal spectrum	94.16
Fourier spectrum	84.93
